# Supplementary material for: In vivo chromatic and spatial tuning of foveolar retinal ganglion cells in Macaca fascicularis
Source: PLoS One. 2022 Nov 29;17(11):e0278261. doi: 10.1371/journal.pone.0278261 (PMC9707781; doi:10.1371/journal.pone.0278261)
Supplement: S4 Fig — Observer 1 used the open source software GIMP to segment RGCs in fluorescence images from M2 (large FOV), M3 (large FOV) and M3 (small FOV). The ellipse tool was used to segment the rough boundary of individual RGCs. Observer 2 used the open source software ImageJ to segment the same RGCs in fluorescence images from M2 and M3 using a hand tracing tool to trace the observable edges of each cell’s fluorescence. Under both methods, the area for each cell was computed in terms of pixels2 and then converted to μm2 using the following formula: μmpixel=291.2μmdeg*axiallength24.2mm*FOVwidth496pixels, where 291.2 μm/deg is the human model eye visual angle to retinal extent conversion, 24.2 mm is the human model eye axial length, 496 pixels is the width of the imaging PMT used in the AOSLO system, axial length is the animal’s axial length in mm, and FOV width is the width in degrees of the FOV used. The axial length of animal M2 is 17.2 mm, and the FOV width used was 3.64 deg. The axial length of animal M3 is 16.56 mm, and the FOV widths used were 3.69 deg (large FOV) and 2.54 deg (small FOV). Cells were compared across functional groups identified as L-M/M-L chromatic opponent (L-M), S only responding (S), Luminance only or achromatic (LUM), and mixed L-M/S responses (L-M/S). In (a), the soma areas in μm2 for the four functional groups in M2 are listed as the mean and standard deviation of each group across both observers. In (b), the same comparisons are made for the four functional groups in M3 at the large FOV. In (c), comparisons for the two functional groups found in M3 at the inner edge of the foveal slope (small FOV) are made across both observers. In, (d), summary tables show the p-scores from a Mann-Whitney U test (MATLAB function ranksum(x,y)) comparing the distributions of soma areas across functional groups as measured by both observers separately. All p-values were greater than 0.1, except for Observer 2’s comparison of the L-M group to the S only group in M2 wh [file pone.0278261.s004.pdf]

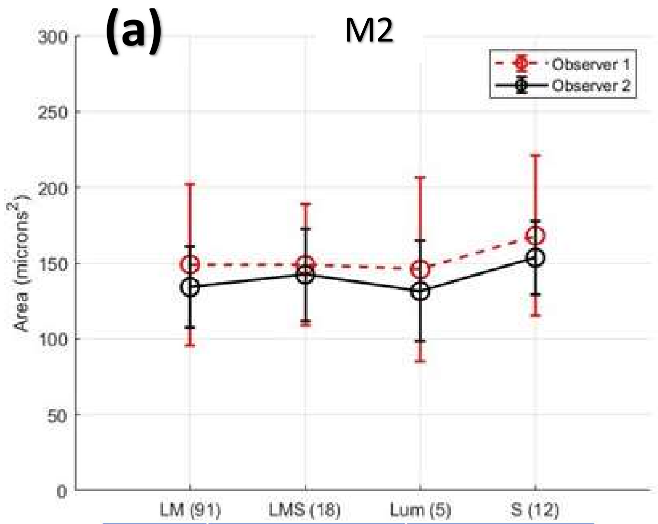

	Mean	SD
Observer 1		
L-M	149.0679	53.3055
L-M/S	148.8017	39.9526
LUM	145.8026	60.7404
S	168.2190	52.6120
Observer 2		
L-M	134.1611	26.9083
L-M/S	142.3934	30.3989
LUM	131.4992	33.2721
S	153.6080	24.1251

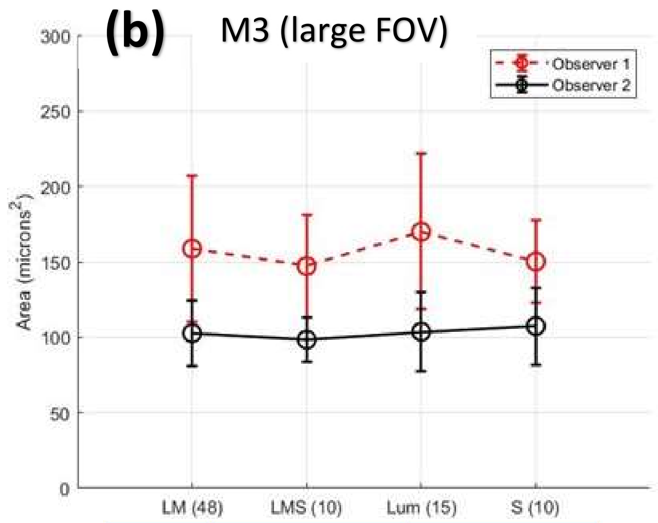

	Mean	SD
Observer 1		
L-M	158.8273	48.3035
L-M/S	147.2437	33.9356
LUM	170.0995	51.4246
S	150.3205	27.0788
Observer 2		
L-M	102.7410	21.9930
L-M/S	98.4555	14.8620
LUM	103.7299	26.2566
S	107.4660	25.6175

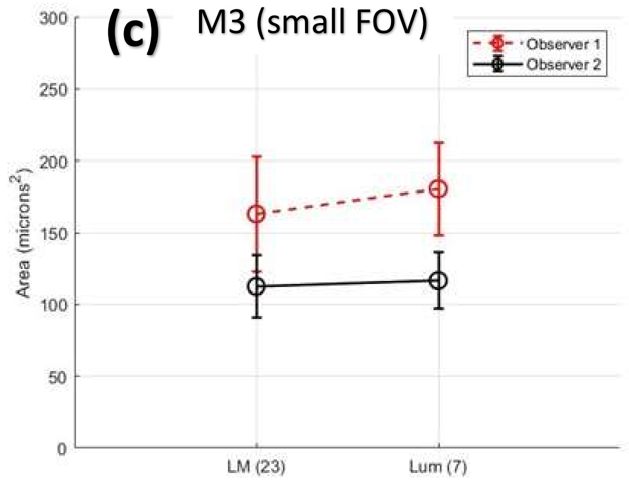

	Mean	SD
Observer 1		
L-M	162.9411	39.6726
LUM	180.5915	32.4137
Observer 2		
L-M	112.5965	21.5090
LUM	116.6258	19.8030

(d)

M2 Large FOV – Observer 1				
	L-M	L-M/S	Lum	S
L-M	-	0.7194	0.7105	0.1442
L-M/S	0.7194	-	0.7093	0.2897
Lum	0.7105	0.7093	-	0.4421
S	0.1442	0.2897	0.4421	-

M2 Large FOV – Observer 2				
	L-M	L-M/S	Lum	S
L-M	-	0.2572	0.6723	0.0118
L-M/S	0.2572	-	0.4103	0.2777
Lum	0.6723	0.4103	-	0.1354
S	0.0118	0.2777	0.1354	-

M3 Large FOV – Observer 1				
	LM	LMS	Lum	S
L-M	-	0.6356	0.2167	1
L-M/S	0.6356	-	0.1827	0.6495
Lum	0.2167	0.1827	-	0.2218
S	1	0.6495	0.2218	-

M3 Large FOV – Observer 2				
	L-M	L-MS	Lum	S
L-M	-	0.7913	0.9475	0.5230
L-M/S	0.7913	-	1	0.4565
Lum	0.9475	1	-	0.6121
S	0.5230	0.4565	0.6121	-

M3 Small FOV – Observer 1	
	L-M
Lum	0.2915

M3 Small FOV – Observer 2	
	L-M
Lum	0.6404

S4 Fig. Soma sizes of RGCs for animals M2 and M3 across chromatic functional groups. Observer 1 used the open source software GIMP to segment RGCs in fluorescence images from M2 (large FOV), M3 (large FOV) and M3 (small FOV). The ellipse tool was used to segment the rough boundary of individual RGCs. Observer 2 used the open source software ImageJ to segment the same RGCs in fluorescence images from M2 and M3 using a hand tracing tool to trace the observable edges of each cell's fluorescence. Under both methods, the area for each cell was computed in terms of pixels^2 and then converted to µm^2 using the following formula: $\frac{\mu m}{pixel} = 291.2\ \frac{\mu m}{deg} * \frac{axial\ length}{24.2\ mm} * \frac{FOV\ width}{496\ pixels}$, where 291.2 µm/deg is the human model eye visual angle to retinal extent conversion, 24.2 mm is the human model eye axial length, 496 pixels is the width of the imaging PMT used in the AOSLO system, axial length is the animal's axial length in mm, and FOV width is the width in degrees of the FOV used. The axial length of animal M2 is 17.2 mm, and the FOV width used was 3.64 deg. The axial length of animal M3 is 16.56 mm, and the FOV widths used were 3.69 deg (large FOV) and 2.54 deg (small FOV). Cells were compared across functional groups identified as L-M/M-L chromatic opponent (L-M), S only responding (S), Luminance only or achromatic (LUM), and mixed L-M/S responses (L-M/S). In **(a)**, the soma areas in µm^2 for the four functional groups in M2 are listed as the mean and standard deviation of each group across both observers. In **(b)**, the same comparisons are made for the four functional groups in M3 at the large FOV. In **(c)**, comparisons for the two functional groups found in M3 at the inner edge of the foveal slope (small FOV) are made across both observers. In, **(d)**, summary tables show the p-scores from a Mann-Whitney U test (MATLAB function ranksum(x,y)) comparing the distributions of soma areas across functional groups as measured by both observers separately. All p-values were greater than 0.1, except for Observer 2's comparison of the L-M group to the S only group in M2 which had p = 0.0118. Based on these results, we cannot reject the hypothesis that the distributions of soma sizes for these functional groups are roughly the same across the two animals measured at the range of eccentricities at which we imaged cell somas.
