# Supplementary figures and images for: In vivo chromatic and spatial tuning of foveolar retinal ganglion cells in Macaca fascicularis
Source: PLoS One. 2022 Nov 29;17(11):e0278261. doi: 10.1371/journal.pone.0278261 (PMC9707781; doi:10.1371/journal.pone.0278261)

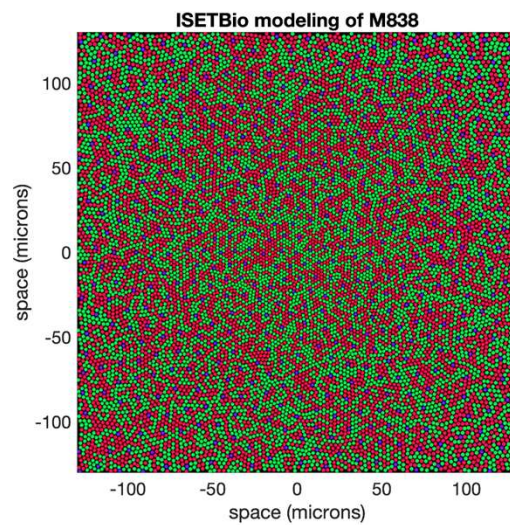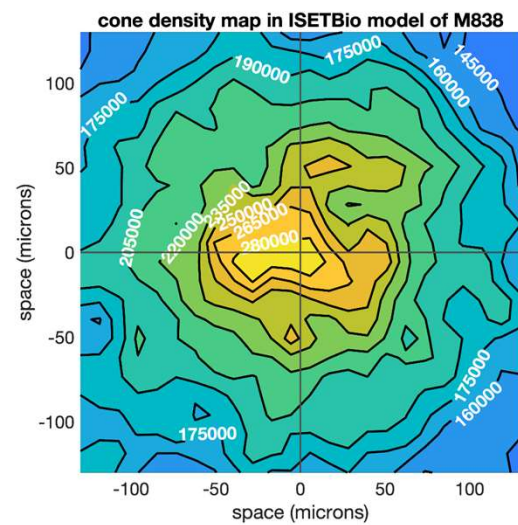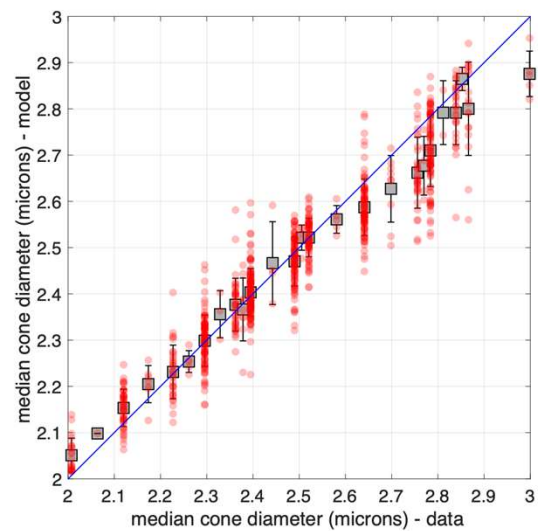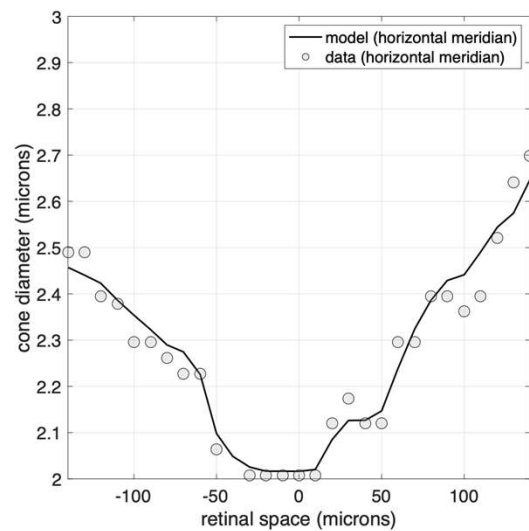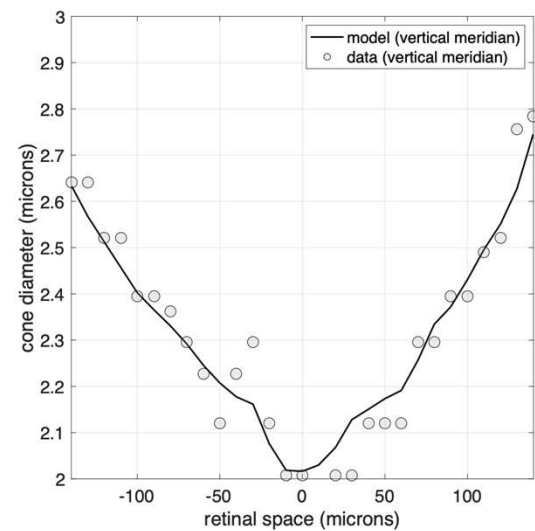

Supplement: S7 Fig — Images showing the performance of the ISETBio cone mosaic generator compared to the actual data from M3. At top, the model trichromatic photoreceptor mosaic generated by ISETBio (left) and the corresponding model cone density (right) (compare to Fig 1B). At bottom left, a comparison of the median diameter of cones are compared between the model and the M3 data. At bottom middle and bottom right, the cone diameters of the model and measured data from M3 are compared across the horizontal and vertical meridians respectively. (PDF) [file pone.0278261.s007.pdf]

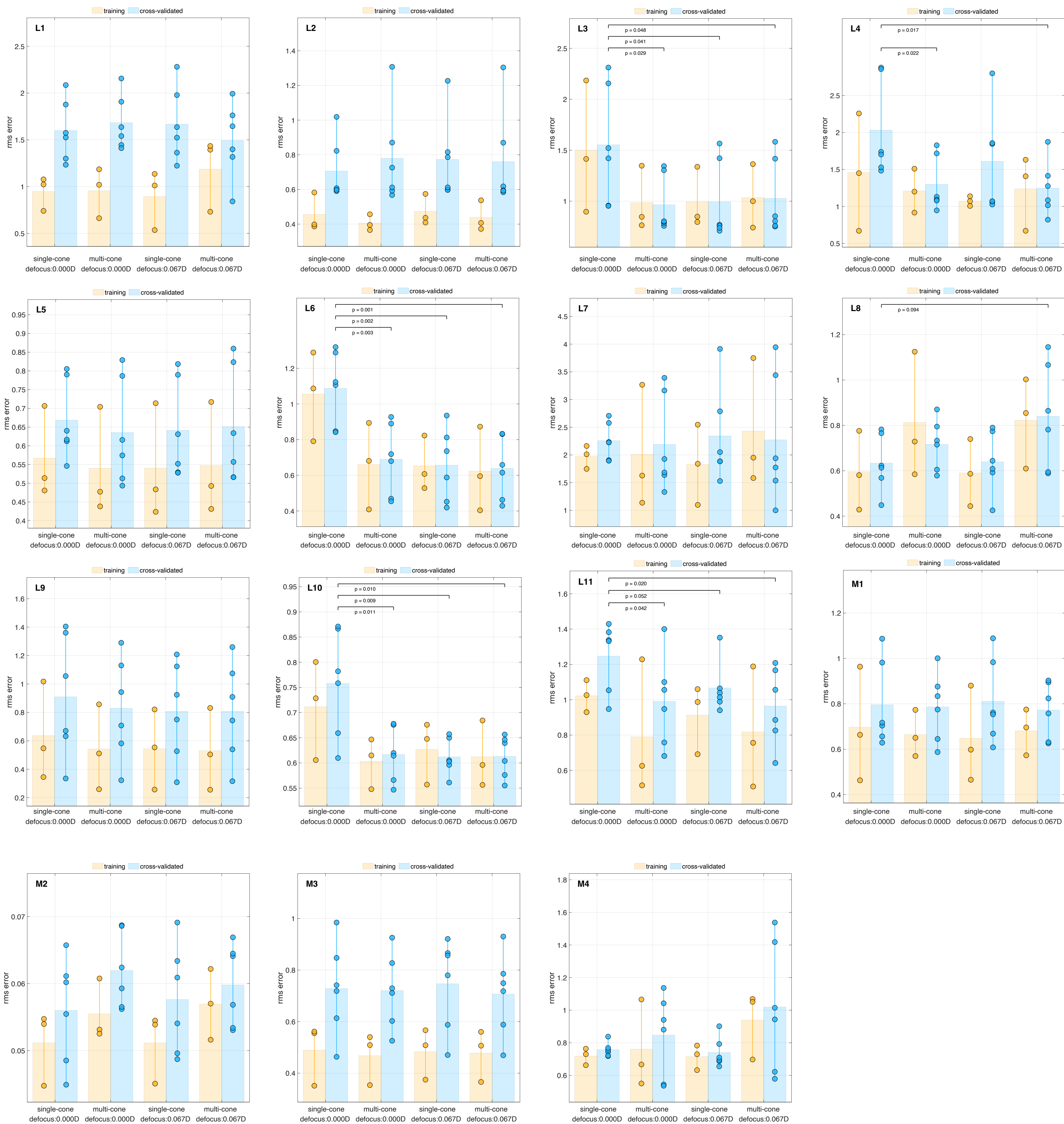

Supplement: S8 Fig — RMS errors for the 4 modeling scenarios we considered are depicted for 12 cells. Yellow and blue bars indicate insample and out-of-sample performance, respectively. During in-sample performance assessment the model is trained and evaluated using data from the same recording session. During out-of-sample performance assessment, the model is trained in one session and evaluated using data from another session. The data do not have enough power to reveal a model with best generalizing (out-of-sample) performance. In a few cells, the 1-cone/0.00D residual defocus model can be ruled out as its performance is significantly worse than the remaining 3. A two sample t-test with unequal variance was used to test against the hypothesis that there is a significant difference in the mean RMS fit errors between 2 modeling scenarios. (PDF) [file pone.0278261.s008.pdf]

single-cone RF  
center model scenario

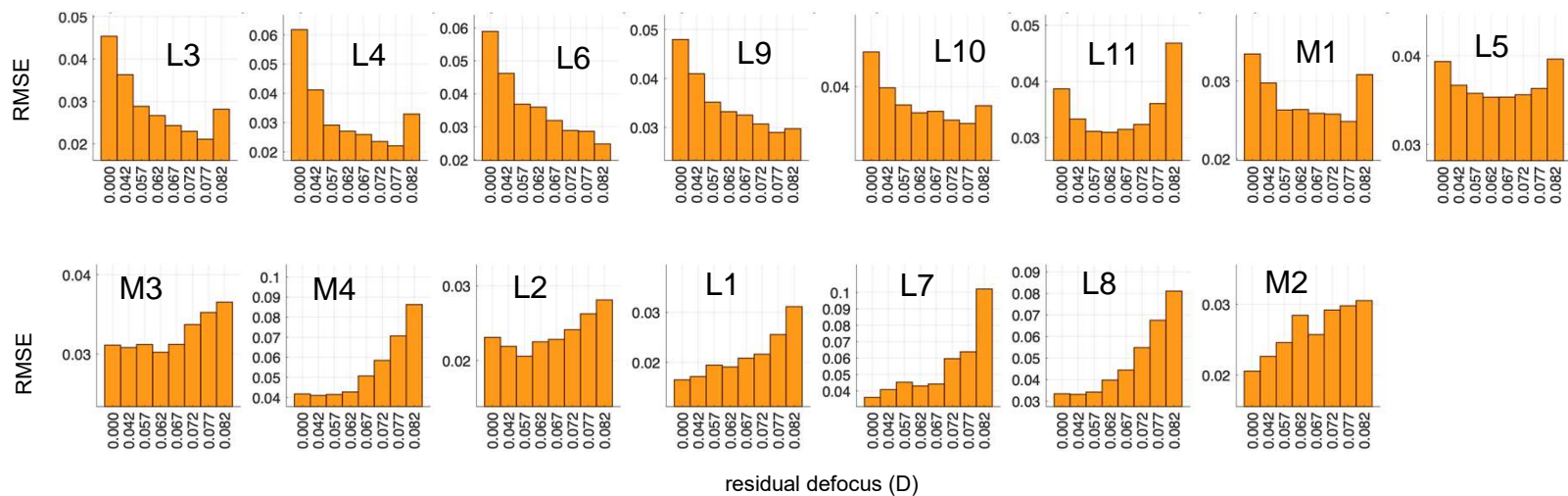

multi-cone RF  
center model scenario

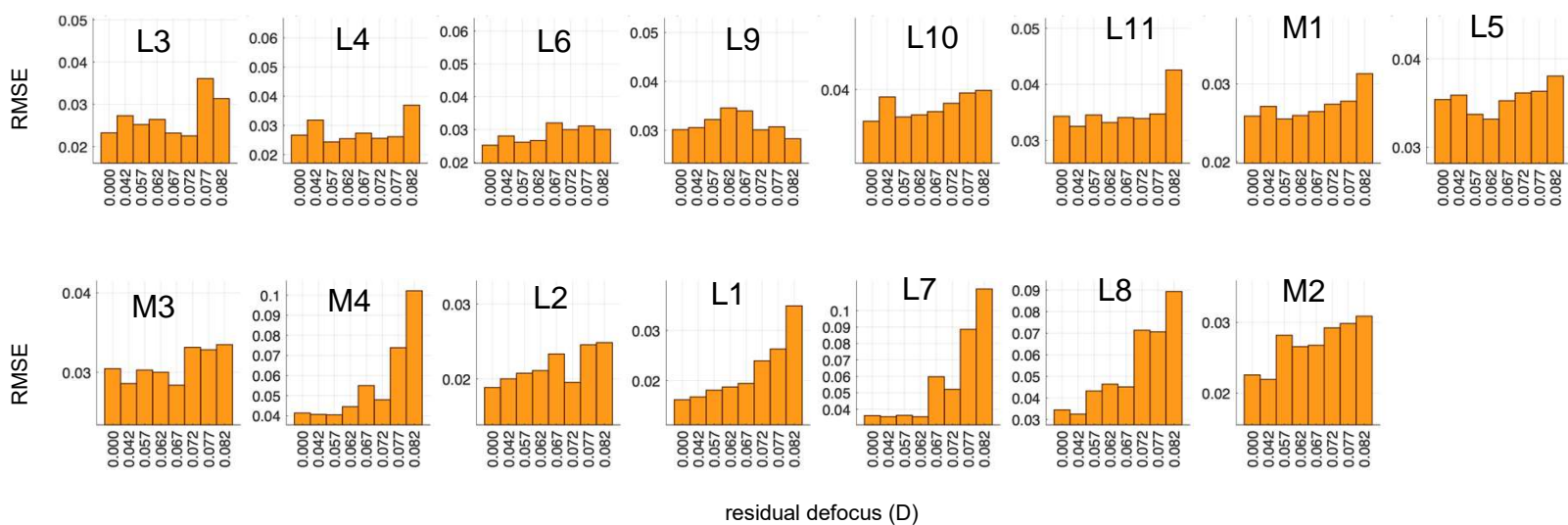

Supplement: S9 Fig — For each cell the optimal residual defocus value for the single cone center and multi-cone center model scenarios was calculated to gauge variability from the chosen 0.067 D reported in the main text. For each cell, the RMSE is shown for various residual defocus values for both model scarios. Cells are labeled L1-11 or M1-4 according to whether we believed they were likely to contain an L cone or M cone at their center. (PDF) [file pone.0278261.s009.pdf]
