# Supplementary material for: In vivo chromatic and spatial tuning of foveolar retinal ganglion cells in Macaca fascicularis
Source: PLoS One. 2022 Nov 29;17(11):e0278261. doi: 10.1371/journal.pone.0278261 (PMC9707781; doi:10.1371/journal.pone.0278261)

single-cone center, 0.00D residual defocus

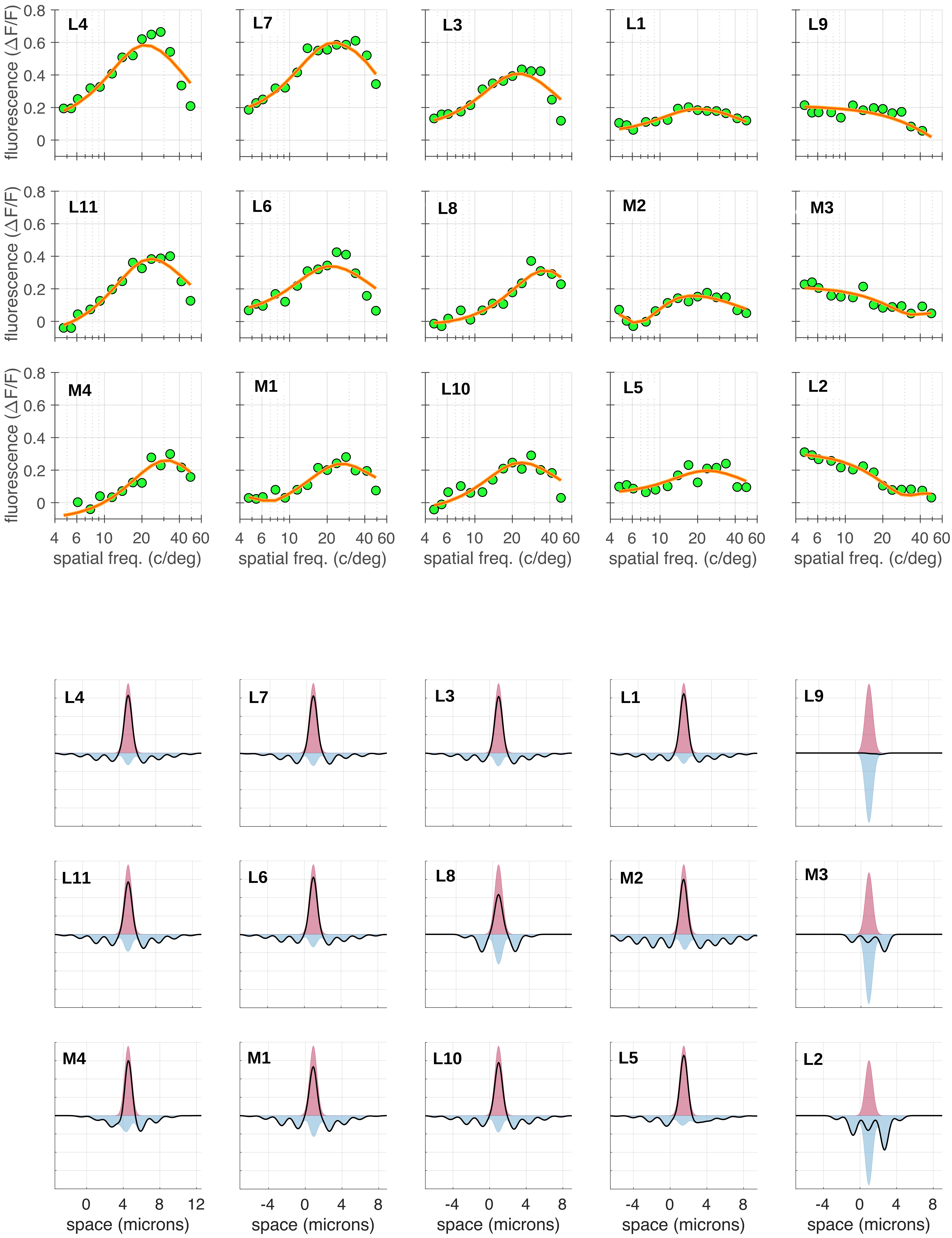

multi-cone center, 0.00D residual defocus

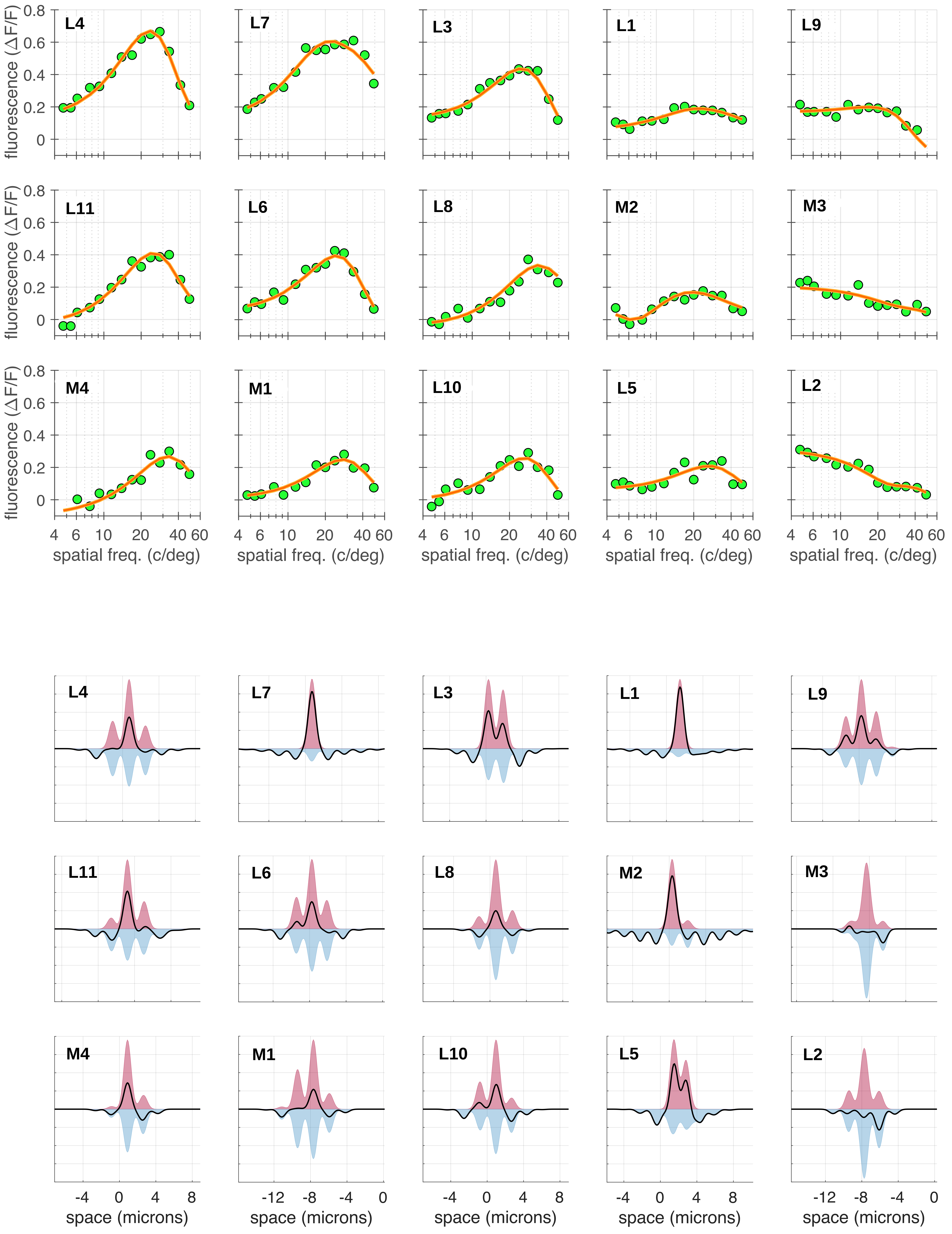

single-cone center, 0.067D residual defocus

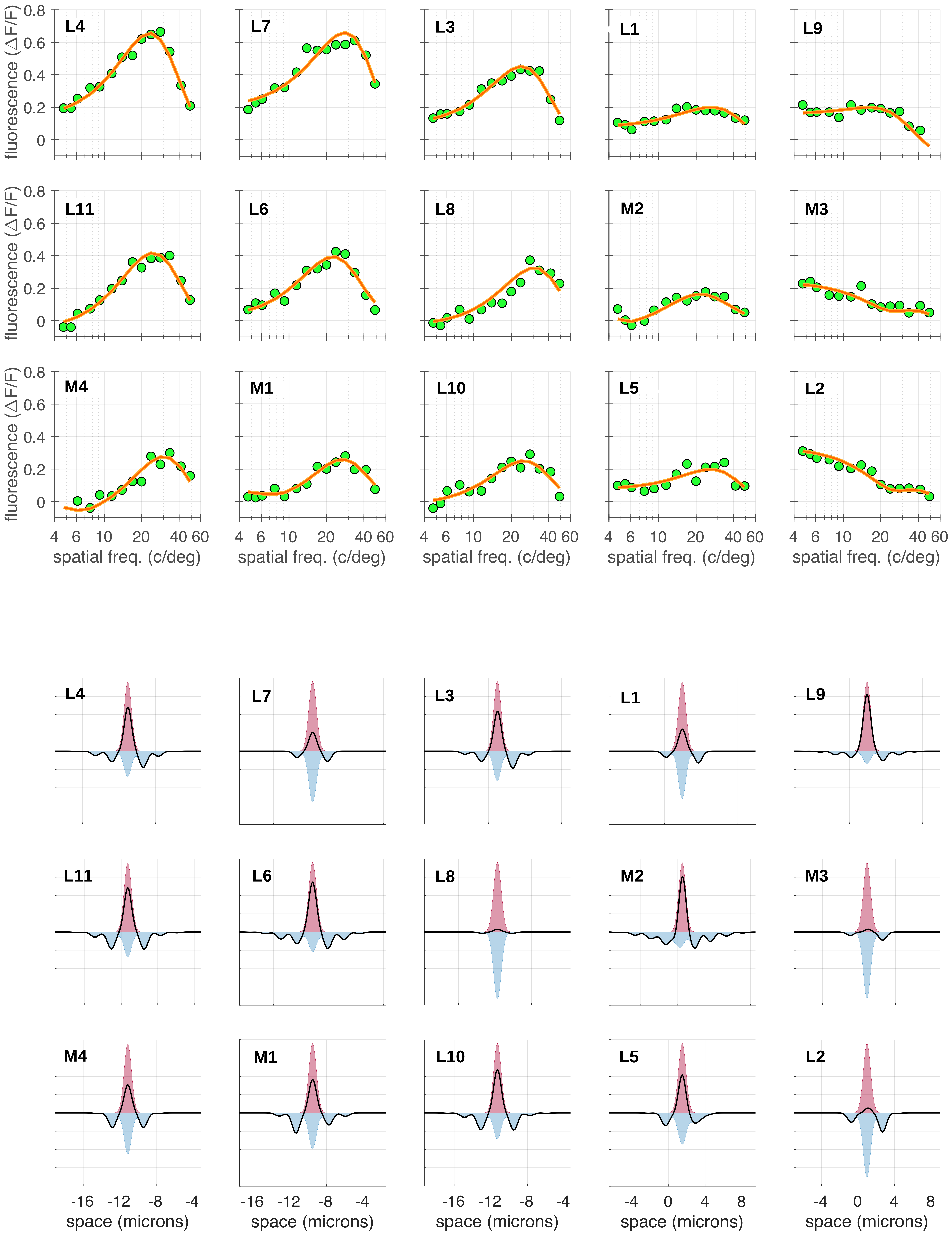

multi-cone center, 0.067D residual defocus

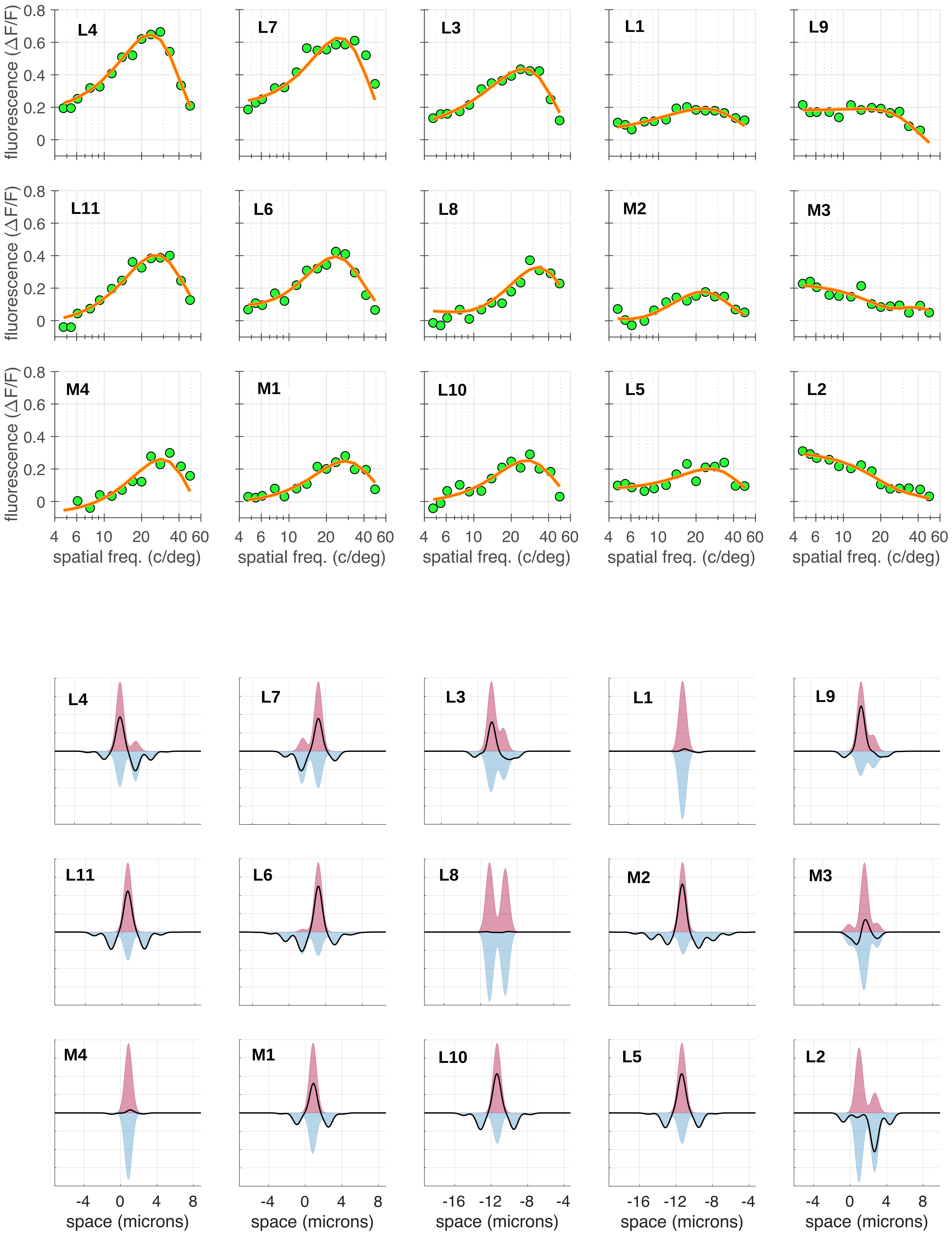

Supplement: S10 Fig — Four model scenarios were considered for all 15 cells: single cone centers with 0 D residual defocus, single cone centers with 0.067 D residual defocus, multi-cone centers with 0 D residual defocus, and multi-cone centers with 0.067 D residual defocus. As can be seen from the fits, the single cone center with 0.067 D defocus performs well for all 15 cells and produces center-surround structures that are consistent with what has been measured from physiology. Cells are labeled L1-11 or M1-4 according to whether we believed they were likely to contain an L cone or M cone at their center. (PDF) [file pone.0278261.s010.pdf]
